# Molecular Evidence of SARS-CoV-2 Virus in Dogs and Cats from Grenada

**DOI:** 10.3390/vetsci12050455

**Published:** 2025-05-09

**Authors:** Vanessa Matthew-Belmar, Trevor Noel, Bhumika Sharma, Katherine Yearwood, Paul Fields, Wayne Sylvester, Nandy Noel, Elsa Chitan, Nikita Cudjoe, Veronica Alexander, Christopher Oura, Calum Macpherson, Andy Alhassan

**Affiliations:** 1 School of Veterinary Medicine, St. George’s University, True Blue, St. George’s, Grenada; vmatthew@sgu.edu (V.M.-B.); bsharma@sgu.edu (B.S.);; 2 Windward Islands Research and Education Foundation, St. George’s University, True Blue, St. George’s, Grenada; trevornoel@sgu.edu (T.N.); nandysnoel@gmail.com (N.N.);; 3 School of Veterinary Medicine, University of West Indies, EWMSC Mt. Hope, St. Augustine, Trinidad and Tobago; 4 School of Public Health and Preventive Medicine, St. Georges University, True Blue, St. Georges, Grenada

**Keywords:** cats, dogs, Grenada, SARS-CoV-2

## Abstract

SARS-CoV-2 is a highly contagious virus that infects humans, wildlife, domesticated and farmed animals. An increase in SARS-CoV-2 variants and human–animal interactions could have implications for the global maintenance and perpetuation of the virus. This study aimed to detect SARS-CoV-2 infection in dogs and cats living in households with and without SARS-CoV-2-positive owners by real-time reverse transcription polymerase chain reaction (qRT-PCR) in Grenada. This cross-sectional study was conducted from August 2020 to April 2022 from dogs (139) and cats (22) in households (96) in Grenada. Nasopharyngeal and oropharyngeal swabs were taken from the dogs and cats to detect SARS-CoV-2. qRT-PCR tests were performed targeting the E and RdRP genes, respectively. Notably, 12% (17/139) of dogs and 23% (5/22) of cats tested positive for SARS-CoV-2. The positive animals were found in 17 (18%) households, all with at least one positive individual. No positive cases of pets were detected in households without infected humans. A statistically significant association (*p* < 0.0001) was observed between humans with SARS-CoV2 and their pets. Phylogenetic tree analysis showed evidence of a relationship between the Grenadian SARS-CoV-2 E gene and other SARS-CoV-2 E gene sequences available in the NCBI database. This study confirmed the concurrent SARS-CoV-2 human/companion animal infection from households in Grenada. Humans and pet animals were positive synchronously; however, the direction of transmission from pets to humans or vice versa remains unknown. This study suggests that pets could play a role in the maintenance, transmission, and prolongation of infection of human-adapted pathogens such as SARS-CoV-2.

## Simple Summary

SARS-CoV-2 is a zoonotic virus that has had a devastating impact on the world, causing high morbidity and variable mortality in human populations. However, the role of domestic animals in the dissemination or maintenance of the virus is poorly understood. This study aimed to detect SARS-CoV-2 infection in dogs and cats living in households with and without SARS-CoV-2-positive owners in Grenada using molecular tools. We found that 12% (17/139) of dogs and 23% (5/22) of cats were infected with SARS-CoV-2. All infected pets were from SARS-CoV-2-positive households, and none of the pets in the SARS-CoV-2-negative households were positive. Sequences from the dog, cat, and human showed similarity to the SARS-CoV-2 E gene genome in the NCBI database. This study provides evidence of infection with SARS-CoV-2 in cats and dogs in Grenada and possible transmission between humans and animals. Positive pets were found in households with confirmed human cases. Continuous monitoring of SARS-CoV-2 in pets remains crucial to understanding the epidemiology of the virus.

## 1. Introduction

Severe acute respiratory syndrome coronavirus 2 (SARS-CoV-2) is a novel RNA virus that can infect humans, wildlife, and farmed and domesticated animals. In humans, a cluster of pneumonia cases was first reported on 31 December 2019, from Wuhan City, Hubei Province, China, and from there, SARS-CoV-2 spread rapidly throughout the world [1]. On 30 January 2020, the World Health Organization (WHO) reported 7834 confirmed cases worldwide and declared the novel coronavirus outbreak a public health emergency of international concern [2]. Considering the alarming rate of SARS-CoV-2 spread and disease severity, the WHO characterized COVID-19 as a pandemic on 11 March 2020; at that time, more than 118,000 cases and 4291 deaths from 114 countries had been reported [3,4].

Several variants of SARS-CoV-2 have emerged since the outbreak of the pandemic [5]. The SARS-CoV-2 Interagency Group (SIG) [6] established a system of four major categories to classify these variants (Class 1 to Class 4). Class one, known as Variant Being Monitored (VBM), comprised the Alpha (B.1.1.7), Beta (B.1.351), Gamma (P.1), Delta (B.1.617.2), Epsilon (B.1.427, B.1.429), Eta (B.1.525), Iota (B.1.526), Kappa (B.1.617.1), Mu (B.1.621), and Zeta (P.2) variants. Class two, known as a Variant of Concern, included the Omicron (B.1.1.529) variant. Class three was designated as a Variant of Interest (VOI) and Class four was classified as a Variant of High Consequence (VOHC). Currently, there are no variants classified under the VOI and VOHC [5]. Some of these variants have been reported to cause infections in both humans and animals [7,8,9].

In Grenada, the first suspected SARS-CoV-2 case in humans was detected using the SARS-CoV-2 antigen test kit on 16 March 2020 from an individual arriving from the United Kingdom [10]. The second SARS-CoV-2-positive individual was on the same flight, and subsequently, several persons on the flight were confirmed positive [11]. The positive cases were confirmed by qRT-PCR in the Caribbean Public Health Agency (CARPHA), WHO/PAHO reference laboratory, Trinidad. During that time in Grenada, the SARS-CoV-2 variants identified were Alpha and Gamma [12]. The first wave of SARS-CoV-2 in Grenada occurred in August 2021 and resulted in over 5000 infections and approximately 200 deaths caused by the Delta variant. In December 2021, the Omicron variant was identified, which was less virulent but more transmissible than the Delta variant. The second wave was due to the Omicron variant in January 2022, with 501 cases per day, and by February 2022, the cases were reduced to 211 per day as a result of government strict control measures, q-RT-PCR testing, and sequencing capabilities on the island [13]. Infected animals may or may not show any clinical signs of infection [14,15,16,17,18]. Recent reports suggest that animals such as cats, dogs, minks, lions, and tigers are naturally susceptible to SARS-CoV-2 infection, while mice, ferrets, hamsters, primates, and tree shrews can be infected experimentally [19,20]. Besides experimental studies, several cases of natural SARS-CoV-2 infection in dogs and cats have been reported [17,18,21,22,23,24,25,26,27,28].

The possibility of the zoonotic spillover of SARS-CoV-2 in humans has also been linked to different live animals that were sold in the Huanan seafood market, such as snakes, frogs, rabbits, marmots, and hedgehogs [19,20]. This has raised concerns regarding the zoonotic potential of these animal species in transmitting SARS-CoV-2 to humans. Furthermore, the reverse zoonotic transmission of SARS-CoV-2 has been suggested in cases where infected humans were in close contact with domestic, farmed, and captive wild animals [29,30,31,32].

Numerous reports indicate that domesticated animals such as cats and dogs contracted SARS-CoV-2 after exposure to infected humans in various regions, including Thailand, the US, Hong Kong, Belgium, Spain, the Netherlands, and Latin America [15,18,33,34,35,36,37].

In Grenada, cats and dogs are popular pets, and many of them live in close contact with their owners, especially during the recent SARS-CoV-2 pandemic. Understanding the potential for SARS-CoV-2 transmission between humans and pets is crucial. The objective of this study was to detect SARS-CoV-2 infection in dogs and cats living in households with and without confirmed SARS-CoV-2 infection in Grenada.

## 2. Materials and Methods

### 2.1. Sample Collection

This study was conducted during the active SARS-CoV-2 diagnosis/surveillance to assess infection in pets (dogs and cats) in 96 households (HHs) with and without SARS-CoV-2 in Grenada from August 2020 to April 2022. The human households’ samples were collected and tested by the National Testing team, Ministry of Health through Windward Islands Research and Education Foundation (WIN-DREF) during the pandemic. The animal study was approved by the St. George’s University (SGU) Institutional Animal Care and Use Committee (IACUC), (SGU IACUC-21003-R), Institutional Review Board (IRB) (SGU IRB-24051), and the Ministry of Health in Grenada. Written consent was obtained from all pet owners enrolled in this study. The animals selected in the study had no clinical signs suggestive of SARS-CoV-2 and were in close contact with their owners. Nasopharyngeal (NP) and oropharyngeal (OP) swabs were collected from a total of 161 pets (139 dogs and 22 cats) by a veterinarian in Grenada. The animal test was performed at the School of Veterinary Medicine diagnostic laboratory.

### 2.2. RNA Isolation and PCR Amplification

Nucleic acid extraction: Total ribonucleic acid (RNA) was extracted from 200 µL of nasopharyngeal and oropharyngeal swab samples using the Quick-RNA Viral Kit (ZYMO Research, Irvine, CA, USA), following the manufacturer’s instructions. RNA was eluted into 30 µL of sterile RNAse, DNAse-free water and immediately subjected to quantitative reverse transcriptase polymerase chain reaction (qRT-PCR). A negative extraction control was included with each batch of samples processed.

qRT-PCR assays: Samples were screened through a one-step qRT-PCR assay, targeting the SARS-CoV-2 envelope gene [38]. A commercial kit (LightMix^®^ Modular SARS-CoV-2 (COVID-19) E gene), consisting of primers and probe (tagged with FAM fluorophore), was used for the qRT-PCR assay (TIB Molbiol, Berlin, Germany). Confirmation of the E-gene positives was carried out using LightMix^®^ Modular SARS-CoV-2 (COVID-19) RdRP kit (TIB Molbiol, Berlin, Germany), which amplifies SARS-CoV-2 RNA-dependent RNA polymerase gene (RdRP) using primers and FAM-tagged probes specific for the detection of SARS-CoV-2 [38].

### 2.3. Conventional PCR/Sequencing

The positive samples obtained from the qRT-PCR results were subsequently subjected to conventional RT-PCR to amplify the E and RdRP genes using the extended E gene primers (forward: TTCGGAAGAGACAGGTACGTTAATAGTTA and reverse GACCACATGGAACGCGTACGCGCA) and RdRP gene primers (Forward: GCT CGC AAA CAT ACA ACG and reverse TAAGGAAGGTACACATAATCATCAC) [39] in a conventional RT-PCR (IDT, Coralville, IA 52241, USA). Bidirectional Sanger sequencing was performed to confirm the presence of SARS-CoV-2 and compare human household-positive samples [11].

### 2.4. Data Analysis

Fisher’s Exact test of independence was performed to test for evidence of an association between households with positive human cases and positive pet cases. A 95% level of confidence and a 5% significance level were used. The data analysis was performed using IBM SPSS version 29.

## 3. Results

**Figure 1:**
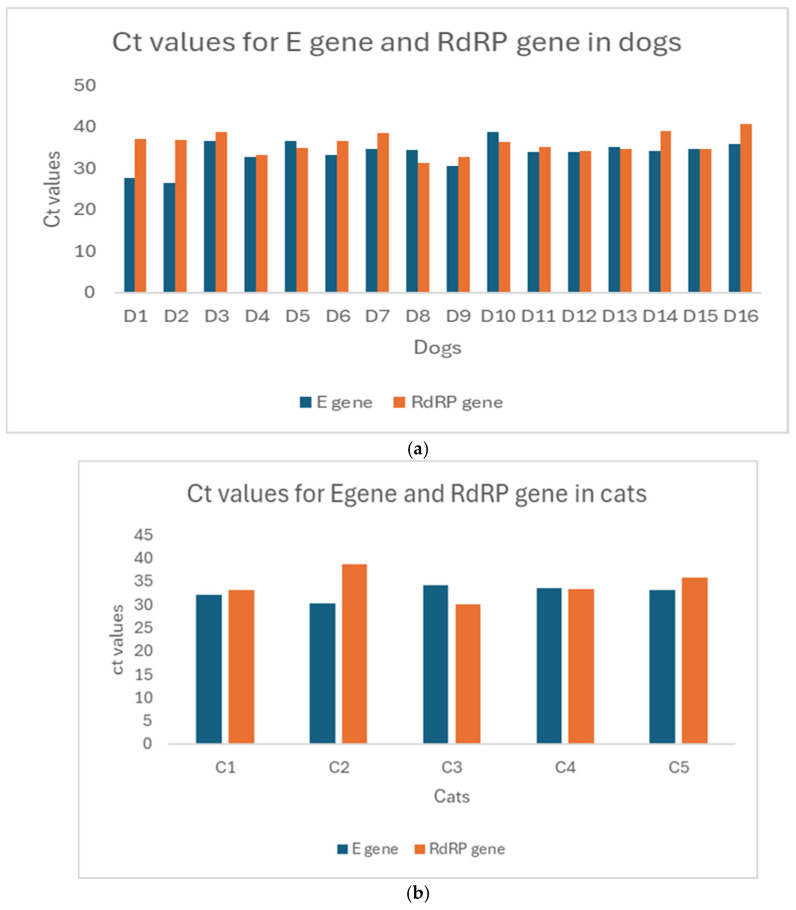
(**a**) Cycle threshold (Ct) values of E. and RdRP genes for dogs tested by qRT-PCR; (**b**) cycle threshold (Ct) values of E and RdRP genes for cats tested by qRT-PCR.

**Figure 2:**
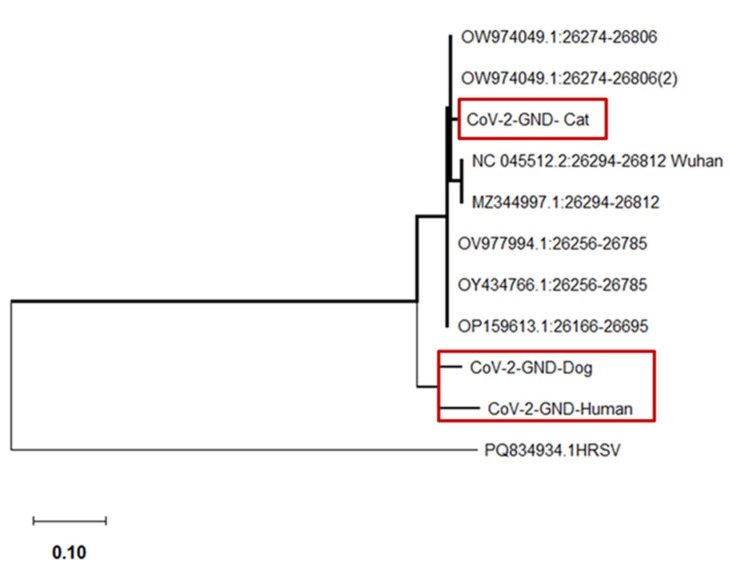
Phylogeny of SARS-CoV-2 E gene in pet dogs, cats, and humans in Grenada. Highlighted in red, represents Grenadian SARS-CoV-2.

A total of 17 (12%) out of 139 dogs and 5 (23%) out of 22 cats were positive for SARS-CoV-2 RNA confirmed by qRT-PCR (Table 1). SARS-CoV-2-infected pets were found in 17 (18%) out of the 96 households tested in this study (Table 1). Notably, 7 out of the 96 (7%) households had both cats and dogs living together, and of those, 2 (29%) were positive for SARS-CoV-2 (Table 2). In these two households, six pets (three cats and three dogs) tested positive for SARS-CoV-2 (Table S1). As shown in Table 2, all SARS-CoV-2-positive cats and dogs were detected in households confirmed to be positive for SARS-CoV-2. A statistically significant association (*p* < 0.0001) was observed between humans with SARS-CoV-2 and their pets (Table 2). In the SARS-CoV-2-positive dogs, the Ct values obtained for the E gene were 26.41–38 and 31.20–40.57 for the RdRP gene. In the SARS-CoV-2-positive cats, the Ct values obtained for the E gene ranged between 30.18 and 34.21, and for the RdRP gene, it ranged between 30.12 and 38.63 (Figure 1a,b). Sequence analysis of positive samples (cat, dog, and human) was used for phylogenetic analysis based on the E gene (Figure 2). The phylogenetic tree showed evidence of a relationship between the Grenadian SARS-CoV-2 E gene and other SARS-CoV-2 E gene sequences available in the NCBI database.

Sequences were derived from a cat (CoV-2 GND-cat), dog (CoV-2 GND-dog), and human (CoV-2 GND-human). The evolutionary history was inferred using the maximum likelihood method and the Tamura–Nei model, developed by Tamura K. and Nei M. (1993) [40,41]. The tree is drawn to scale, with branch lengths measured in the number of substitutions per site. There were a total of 1002 positions in the final dataset. Evolutionary analyses were conducted in MEGA X [41].

## 4. Discussion

The current study was conducted to assess the infection among pets living with laboratory-confirmed SARS-CoV-2 infection in households in Grenada. The study results demonstrate SARS-CoV-2 infection in domestic dogs and cats in Grenada with no clinical signs compatible with SARS-CoV-2. SARS-CoV-2 infection in dogs and cats in Grenada appeared to be associated with the SARS-CoV-2-positive status of infected households. As shown in previous reports, higher cycle threshold (Ct) values represent a low viral load and a lower risk of infection and transmission in infected individuals [42,43,44,45,46]. In this study, pet samples with Ct values of ≤38 were considered positive for the E gene, while samples with a Ct value of ≤42 were considered positive for the RdRP gene. Moreover, Ct values below 20 have been associated with high viral load, replication, and severity of infection [47]. In this study, the Ct values for both the E and RdRP genes in cats and dogs were 26 and above, showing low viral load. Additionally, it is uncertain whether the humans were infecting the dogs and cats or vice versa. While the evidence for human-to-human transmission has been reported, epidemiological studies indicate that dogs and cats can contract the virus from infected owners [35,48,49]. Our data indicate that the SARS-CoV-2-infected companion animals identified in this study had been in contact with an infected individual within the same household. This suggests that people, particularly pet owners, are likely the source of infection for their pets.

A study in Thailand confirmed that both a cat and its owner, along with the attending veterinarian, were infected with the same Delta variant of SARS-CoV-2 [18]. In the United States, a pet dog and cat from the same household tested positive for SARS-CoV-2 two days after their owner tested positive [34]. In Hong Kong, 2 out of 15 dogs from households with confirmed human cases of SARS-CoV-2 were found to also be infected with SARS-CoV-2 [15]. A case study in Belgium showed that a cat was infected with SARS-CoV-2 from its owner [36]. In Spain, 1 out of 23 cats tested positive for SARS-CoV-2 and was from an infected household [33]. In the Netherlands, 27 of 156 dogs and 31 of 152 cats were positive for SARS-CoV-2 infection in pets that were in contact with SARS-CoV-2-positive household members [35]. In Latin America, 11 of 42 dogs and 1 of 8 cats tested positive for SARS-CoV-2; these animals were also infected from SARS-CoV-2-positive households [37]. These findings align with our study in Grenada, where the SARS-CoV-2-positive cats and dogs were detected in households with confirmed human infections. Furthermore, evidence of infection showed statistical significance (*p* < 0.0001) between households with humans and pets infected with SARS-CoV-2. However, there are no reported studies on SARS-CoV-2 infections in pet cats and dogs from the Caribbean region, limiting the scope for regional comparison.

During the pandemic, the predominant variant circulating during the first outbreak in Grenada was the Delta variant, followed by the Omicron variant in the affected human population [13]. The initial detection of SARS-CoV-2 in household dogs and cats occurred in February 2022. A limitation of this study was the inability to determine SARS-CoV-2 variants in the positive dog and cat samples. Additionally, whole-genome sequencing was not possible since the weight of the positive PCR products was low, and the variant was unable to be determined due to poor genome coverage with high Ct values [49]. Subsequently, conventional PCR was performed on the positive samples targeting both the E and RdRP genes. However, phylogenetic tree analysis showed evidence of a relationship between the Grenadian SARS-CoV-2 E gene and other SARS-CoV-2 E gene sequences available in the NCBI database.

While the duration of SARS-CoV-2 viral shedding in dogs and cats has been documented in Iran [48], Brazil (11 to 51 days), China (28 days), and the United States (32 days) [34,44,50,51], this study was unable to determine the shedding of the virus in the infected pets due to limitation in laboratory facilities and government restrictions.

## 5. Conclusions

This is the first study that established evidence of SARS-CoV-2 infection in cats and dogs from SARS-CoV-2-positive households in Grenada during the pandemic. These findings correspond with previous reports of SARS-CoV-2 infection in various animal species, highlighting the need to take proactive measures to prevent infections in pets. In Addition, this study provides evidence of pet infections, reinforcing the necessity of active surveillance within pet populations. It further emphasizes the significance of the One Health, One Medicine approach in monitoring outbreaks like the SARS-CoV-2 pandemic, as pets could play an important role in the transmission and persistence of zoonotic pathogens.

## Figures and Tables

**Table 1:** Distribution of SARS-CoV-2 status in pet dogs and cats.

	Animal Status		
Species	Negative	Positive	Total
Cat	17 (77%)	5 (23%)	22
Dog	122 (88%)	17 (12%)	139
Total	139	22	161

**Table 2:** Evidence of association between humans and their pets and whether or not they test positive for SARS-CoV-2 (*p*-value < 0.0001).

96 Households (HH)	COVID Positive Cats	COVID Positive Dogs	COVID Negative Cats	COVID Negative Dogs	Total Number of COVID Positive Animals	Total Number of Animals both Positive and Negative
17 HH with COVID-19-positive humans	5	17	0	0	22	22
79 HH with COVID-19-negative humans	0	0	17	122	0	139
	5 Total positive cats	17 total positive dogs	17 total negative Cats	122 total negative dogs	22	161

## Data Availability

The data presented are available within this study.

## Supplementary Materials

The following supporting information can be downloaded at: https://www.mdpi.com/article/10.3390/vetsci12050455/s1, Table S1: Distribution of SARS-CoV-2 among positive and negative households and their pets.
