# Supplementary material for: Molecular Evidence of SARS-CoV-2 Virus in Dogs and Cats from Grenada
Source: Vet Sci. 2025 May 9;12(5):455. doi: 10.3390/vetsci12050455 (PMC12115539; doi:10.3390/vetsci12050455)
Supplement: Supplementary file 1 [file vetsci-12-00455-s001.zip › vetsci-3509567-supplementary.pdf]

Table S1: Distribution of SARS-CoV-2 among positive and negative households and their pets

Source	Positive for SARS-CoV-2		Negative for SARS-CoV-2	
	Households	Total number	Households	Total number
Dogs	13	14	66	111
Cats	2	2	8	9
Dogs and Cats	2	6	5	19
**Total pet animals**	**17**	**22**	**79**	**139**
**Total Humans**	**17**	**17**	**79**	**79**
